# CSCD2: an integrated interactional database of cancer-specific circular RNAs

**DOI:** 10.1093/nar/gkab830

**Published:** 2021-09-22

**Authors:** Jing Feng, Wenbo Chen, Xin Dong, Jun Wang, Xiangfei Mei, Jin Deng, Siqi Yang, Chenjian Zhuo, Xiaoyu Huang, Lin Shao, Rongyu Zhang, Jing Guo, Ronghui Ma, Juan Liu, Feng Li, Ying Wu, Leng Han, Chunjiang He

**Affiliations:** School of Computer Science, Wuhan University, Wuhan430072, China; School of Basic Medical Sciences, Wuhan University, Wuhan430071, China; School of Basic Medical Sciences, Wuhan University, Wuhan430071, China; School of Basic Medical Sciences, Wuhan University, Wuhan430071, China; School of Basic Medical Sciences, Wuhan University, Wuhan430071, China; School of Basic Medical Sciences, Wuhan University, Wuhan430071, China; School of Basic Medical Sciences, Wuhan University, Wuhan430071, China; College of Biomedicine and Health, Huazhong Agricultural University, Wuhan430070, China; School of Basic Medical Sciences, Wuhan University, Wuhan430071, China; School of Basic Medical Sciences, Wuhan University, Wuhan430071, China; College of Biomedicine and Health, Huazhong Agricultural University, Wuhan430070, China; School of Basic Medical Sciences, Wuhan University, Wuhan430071, China; College of Biomedicine and Health, Huazhong Agricultural University, Wuhan430070, China; School of Computer Science, Wuhan University, Wuhan430072, China; School of Basic Medical Sciences, Wuhan University, Wuhan430071, China; School of Basic Medical Sciences, Wuhan University, Wuhan430071, China; Center for Epigenetics and Disease Prevention, Institute of Biosciences and Technology, Texas A&M University, Houston, TX77030, USA; School of Basic Medical Sciences, Wuhan University, Wuhan430071, China; College of Biomedicine and Health, Huazhong Agricultural University, Wuhan430070, China

## Abstract

The significant function of circRNAs in cancer was recognized in recent work, so a well-organized resource is required for characterizing the interactions between circRNAs and other functional molecules (such as microRNA and RNA-binding protein) in cancer. We previously developed cancer-specific circRNA database (CSCD), a comprehensive database for cancer-specific circRNAs, which is widely used in circRNA research. Here, we updated CSCD to CSCD2 (http://geneyun.net/CSCD2 or http://gb.whu.edu.cn/CSCD2), which includes significantly more cancer-specific circRNAs identified from a large number of human cancer and normal tissues/cell lines. CSCD2 contains >1000 samples (825 tissues and 288 cell lines) and identifies a large number of circRNAs: 1 013 461 cancer-specific circRNAs, 1 533 704 circRNAs from only normal samples and 354 422 circRNAs from both cancer and normal samples. In addition, CSCD2 predicts potential miRNA–circRNA and RBP–circRNA interactions using binding motifs from >200 RBPs and 2000 microRNAs. Furthermore, the potential full-length and open reading frame sequence of these circRNAs were also predicted. Collectively, CSCD2 provides a significantly enhanced resource for exploring the function and regulation of circRNAs in cancer.

## INTRODUCTION

As an important covalently closed RNA, circular RNAs (circRNAs) have been largely discovered by high-throughput sequencing in many species (1). circRNAs are generated by back-splicing of a linear gene (2), function as sponges of microRNA (3) and/or RNA-binding protein (4) and may also be translated into small peptides (5). Previous work demonstrated that circRNAs play important roles in human diseases, especially in cancer (6). Hundreds of circRNAs are involved in human epithelial–mesenchymal transition (EMT) (7), and endogenous circRNAs with 16–26 bp imperfect RNA duplexes can regulate innate immunity by acting as inhibitors of double-stranded RNA (dsRNA)-activated protein kinase (PKR) (8). Due to their resistance to nucleic acid exonuclease and long half-lives, circRNAs can also serve as potential diagnostic markers (9). To better utilize the resources of circRNAs from different samples, we constructed a database named CSCD to characterize cancer-specific circRNAs based on cancer and normal cell lines in 2018 (10). CSCD is a popular database and attracted wide attention in circRNA field. Compared to other resources such as circRic (11) and miOncocirc (12), CSCD focused on identifying cancer-specific circRNAs that are not presented in normal tissues/cell lines. With the significantly increased sequencing datasets from cancer and normal samples released in the last 3 years, an updated resource for cancer-specific circRNAs is necessary. In CSCD2, we collected >1000 samples, which is five times larger than CSCD, and we identified >1 million more circRNAs than CSCD. Furthermore, an interactive interface is provided for users to view circRNA–miRNA or circRNA–RBP interaction based on potential interactions from the new results. The full-length and open reading frame (ORF) sequences of each circRNA are also predicted according to their parent sequences, which is important for circRNA functional research (13,14). Collectively, CSCD2 is a comprehensive resource for cancer-specific circRNAs with enhanced functional modules, which can significantly contribute to circRNA research in cancer.

## DATA SUMMARY AND METHODS

### Sample collection

**Figure 1.**
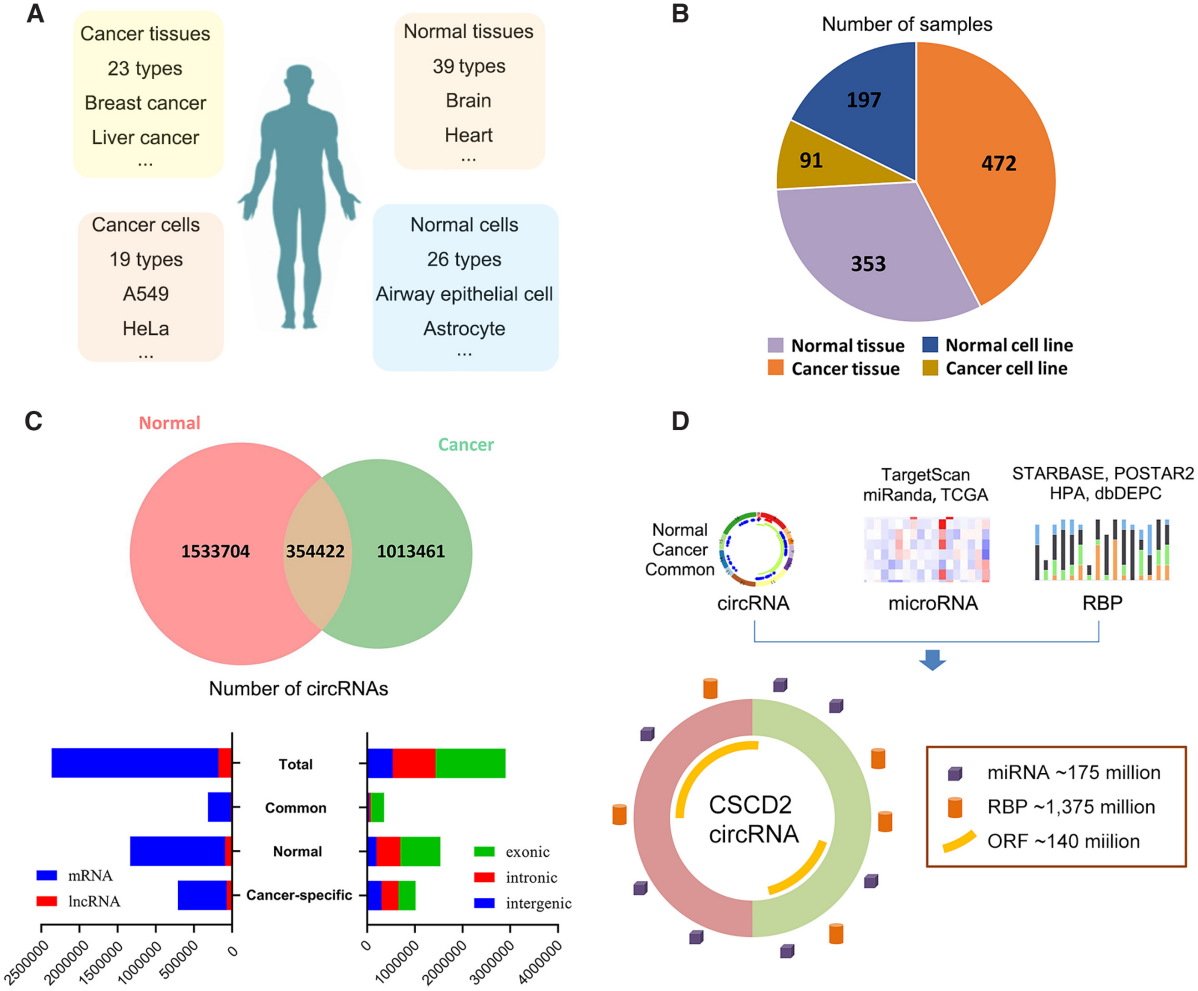
Overview and content of CSCD2. (**A**) Number of tissues or cell types collected in CSCD2. (**B**) Number of samples from cell lines or tissues included in CSCD2. (**C**) Number of circRNAs identified in cancer samples, normal samples, or both tumor and normal samples. CircRNAs are classified according to the attribute of host genes (e.g. mRNA or lncRNA) or the genomic locations (e.g. exonic, intronic or intergenic). (**D**) CSCD2 offers ∼2.9 millions of circRNAs and potential interactive miRNAs and RBPs, as well as their expression level in cancer samples.

We collected RNA-Seq datasets of 1113 samples from tissues or cell lines from the ENCODE (https://www.encodeproject.org/) and SRA (https://www.ncbi.nlm.nih.gov/sra/) databases, including 472 cancer tissue samples, 353 normal tissue samples, 91 cancer cell line samples and 197 normal cell line samples (Figure 1A and B, Supplementary Table S1). The RNA-Seq libraries were prepared from total RNA with the rRNA depleted or the polyA minus (polyA-) enriched method.

### Identification of cancer-specific circRNAs

After trimming the adapter and low quality bases, reads from RNA-Seq were mapped to the human genome (genome assembly GRCh38), and then circRNAs were identified by four algorithms: CIRI2 (15), circRNA_finder (16), find_circ (2) and circexplorer2 (17). GENCODE (version 32) and Refseq (version GCF_000001405.39) gene annotation was used to annotate circRNAs. We included all circRNAs (BSJ ≥ 1) identified by either one of four algorithms. SRPTM (number of circular reads / number of mapped reads (units in trillion) / read length) in previous work (10) was used to normalize the expression levels of circRNAs. A 2 bp mismatch of coordinates was tolerated by merging the same circRNAs in different algorithms and samples.

### Prediction of interactions between microRNAs and circRNAs

To reveal the potential interactions between microRNAs and circRNAs, a 100 bp window (±50 bp), as previously described (11), surrounding the back-splice site of each circRNA (which is possibly referring to the circRNA-specific regions, not linear RNA) was selected to scan the potential miRNA response elements (MREs) using TargetScan (18) and miRanda (19). A total of 2064 microRNAs were investigated in the prediction. The expression of microRNA in 32 types of cancer were extracted from TCGA database (https://cancergenome.nih.gov).

### Prediction of the interactions between RBPs and circRNAs

Integrated CLIP-Seq data were downloaded from STARBASE (20) and POSTAR2 (21) which included protein binding sites of 207 RBPs, and we were able to identify potential RBP-binding events in circRNAs. In addition, we integrated the IHC staining data and mass spectrometry data from HPA (22) and dbDEPC (23) for each RBP in CSCD2 to characterize the expression of circRNA-interacted RBPs across different cancer types.

### Prediction of full length and ORFs

R package FcircSEC (24) was used to extract the full length of circRNAs with the human reference genome (Hg38) as the reference. To examine the translational potential of circRNAs, the ORFs were predicted using getORF from EMBOSS (http://emboss.open-bio.org/) with parameters, -minsize 75 and -circular Y. The minimal ORF length was set as 75 nt according to a previous study (25).

### Database implementation

**Figure 2.**
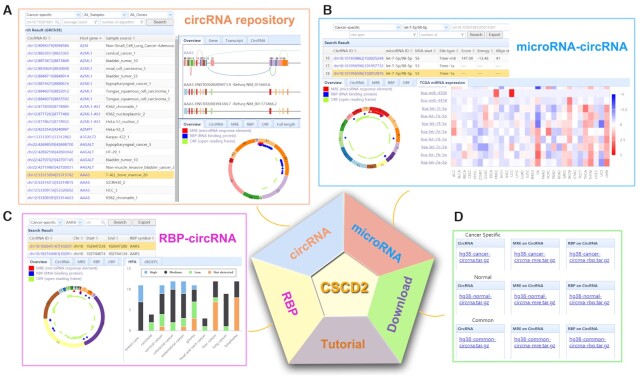
Interface of CSCD2. (**A**) Panel of circRNA repository. circRNA can be viewed and searched by sample name, gene symbol and circRNA ID. The information about the genes, transcripts and circRNAs are displayed on upper right panel. The information of circRNA and related location of MREs, RBPs and ORFs are displayed on lower right panel. (**B**) Panel of microRNA–circRNA interaction. microRNA-associated circRNAs can be viewed and searched by sample type and microRNA ID. The information about the circRNAs and the related locations of MREs, RBPs and ORFs are displayed on lower left panel. The expression level of microRNAs from TCGA cancer samples are displayed on lower right panel. (**C**) Panel of RBP-circRNA interaction. RBP-associated circRNAs can be viewed and searched by sample type and RBP gene symbol. The information about the circRNAs and the related locations of MREs, RBPs and ORFs are displayed on lower left panel. The expression level of the selected RBP from HPA and dbDEPC are displayed on lower right panel. (**D**) Download panel. All files including cancer or normal-enriched circRNAs, interactive microRNAs and RBPs can be downloaded through the Download page.

All the results, including gene annotations, circRNAs, MREs, RBPs, ORFs and full length associated with circRNAs, were implemented into a set of interactive MySQL tables. ThinkPHP, an open-source web framework based on a PHP (https://github.com/top-think) and JavaScript library, were used to construct the CSCD2 database. The web interface of CSCD2 is summarized in Figure 2. The main function of CSCD2 is composed of three pages: circRNA, microRNA and RBP.

## IMPROVED CONTENT AND NEW FEATURES

### Database content

A total of 2 901 587 circRNAs were identified and included in CSCD2. Among these, 1 013 461, 1 533 704 and 354 422 circRNAs were identified only in cancer samples (CS-circRNA), normal samples, and both cancer and normal samples (common circRNAs), respectively. Among the total circRNAs, 1 465 746, 900 421 and 535 420 circRNAs were located in exonic, intronic and intergenic regions, respectively. The full-length sequences of all circRNAs are also predicted. We identified 2 185 161 and 181 006 circRNAs located in mRNA and lncRNA, respectively (Figure 1C and Supplementary Table S2). We identified 64 125 092, 92 809 837 and 18 368 820 MREs in CS-circRNAs, normal circRNAs and common circRNAs, respectively, through integrating the results of targetscan and miRanda. Furthermore, 339 024 277, 744 504 232 and 292 022 461 RBP binding sites were identified in CS-circRNAs, normal circRNAs and common circRNAs, respectively. We also identified a total of 8 884 187, 18 539 669 and 3 749 154 ORFs in cancer-specific, normal and common circRNAs, respectively (Figure 1D and Supplementary Table S2).

### Database access

CSCD2 includes three user-friendly web interfaces. In circRNA panel (Figure 2A), users can browse circRNAs by selecting the sample type, sample name and gene symbol and can search by circRNA ID (e.g. chr12:53310577|53311003), which represents the donor and acceptor sites of each circRNA or gene symbol in the search box. All information, including the circRNA ID, parent gene symbol, UCSC genome browser link, sample type for each circRNA, is displayed in the table. The filtration and sort function are also provided for users to filter the table by read counts, number of algorithms, genomic region and to sort by columns. The gene symbol links to the upper right panel with all circRNAs across different samples. The circRNA ID links to the lower right panel with a circRNA in a specific sample. In the upper right panel, users can view the circRNAs and their linear parent genes in the overview tab. Linear gene structures are displayed as different colored rectangles for exons, black lines for introns and colorful curves for circRNAs. All transcripts from Refseq and Gencode database of parent genes are also displayed below as annotation. Users can zoom in for a high-resolution image by clicking the top right corner of the panel. All the detailed information is listed in the gene, transcript and circRNA tabs. The circRNA curve links to specific circRNA in the lower right panel. Users can view selected circRNAs consisting of exons in a colored circle. Each arc with a numeric ID depicts one exon, while introns are displayed in black lines. Users can also view the number and position of the MRE (red triangle), RBP (blue rectangle) and ORF (green arc) elements located in circRNA and check the detailed information through the circRNA, MRE, RBP and ORF tabs, respectively. Clicking the miRNA or RBP name in the table could jump to the miRNA or RBP page and allow users to view all circRNAs binding by this miRNA or RBP.

In microRNA panel (Figure 2B), users can browse microRNAs by selecting the sample type or selecting/inputting microRNA ID (e.g. miR-637). All information, including the circRNA ID, microRNA ID, MSA start (Seed start), MSA end (Seed end), site type, score, energy, align start (miRNA start), align end (miRNA end), and algorithm is displayed in the table. Users can select multiple miRNAs and click ‘Search’ button to obtain all circRNAs binding by multiple miRNAs. CircRNA ID links the right panel with a circRNA in a specific sample. The upper right panel displays the selected circRNAs that consist of exons in a colored circle. Users can also view the number and position of MREs (red triangle), RBPs (blue rectangle) and ORFs (green arc) located in circRNA and check the detailed information through the circRNA, MRE, RBP and ORF tabs, respectively. In the lower right panel, users can view the expression status of selected microRNA in different cancers. The detailed information is listed in the TCGA microRNA expression tab.

In RBP panel (Figure 2C), users can browse RBPs by selecting a sample type or selecting/inputting RBP gene symbol. All information, including the circRNA ID, genomic coordinates of the RBP (chromosome, start and end) and RBP gene symbol, is displayed in the table. Users can also select multiple RBPs and click ‘Search’ button to obtain the circRNAs binding by those selected RBPs. The circRNA ID links to the upper right panel with a circRNA in a specific sample. In the upper right panel, users can view selected circRNAs consisting of exons in a colored circle. Users can also view the number and position of MREs (red triangle), RBPs (blue rectangle) and ORFs (green arc) located in the circRNA and view the detailed information through the circRNA, MRE, RBP and ORF tabs, respectively. In the lower right panel, users can view the expression status of selected RBPs in different cancers. The detailed information is listed in the HPA and ORF tabs. Four degrees of IHC staining (high, medium, low and not detected) are displayed for each RBP. All files including cancer or normal-enriched circRNAs, interactive microRNAs and RBPs can be downloaded through the Download page (Figure 2D).

## SUMMARY AND FUTURE PERSPECTIVES

As a large number of new datasets are available recently, we updated our CSCD database to CSCD2, an integrated interactional database with more samples and new functional modules. CSCD2 have significant updates compare to CSCD, and it has unique feature compared to other circRNA databases, including circRic (11), circAtlas (14) and circBase (26) (Supplementary Table S3). We highlighted three important upgrades in CSCD2: first, it includes a large number of samples (>1000), including ∼800 tissue samples and ∼300 cell line samples, which is about five times more than CSCD. With these new data resources, we were able to identify significantly larger number of circRNAs (∼2.9 million). Second, the interactions of circRNA–microRNA and circRNA–RBP are predicted and indexed. Users can search microRNAs/RBPs to acquire the interacted circRNAs or vice versa to search circRNAs to acquire microRNAs/RBPs. The expression of microRNAs and RBPs are also provided in CSCD2 for further evaluation. Third, due to the functional significance of the full length of circRNAs and the translational ability of circRNAs, we added additional information including full length and ORF sequence of circRNAs in CSCD2.

In summary, CSCD2 is an updated database for investigating the potential function of cancer-specific circRNAs with significant larger number of samples and circRNAs, as well as new functions to better interpret the functions of circRNAs, including the interactions of circRNA–microRNA and circRNA–RBP, full length of circRNAs and the translational ability of circRNAs. Considering that there is debate over the functional significance of circRNAs (27), it is possible that some circRNAs are not functional, but many of them are still functionally significant. Our CSCD2 provides a unique platform for further investigation of the functions of circRNAs in cancer. With the development of more advanced technologies, especially the application of nanopore sequencing in circRNA research (28), we will regularly update the database with new data and functions.

## Supplementary Material

gkab830_Supplemental_Files
